# A single nucleotide polymorphism produces different transcription profiles in *Campylobacter jejuni’s cysM*

**DOI:** 10.3389/fmicb.2025.1501331

**Published:** 2025-03-21

**Authors:** Nereus W. Gunther, Siddhartha Kanrar, Aisha Abdul-Wakeel, Michael J. McAnulty, John Renye, Joseph Uknalis, Gaylen A. Uhlich

**Affiliations:** ^1^Characterization and Interventions for Foodborne Pathogens Research Unit, United States Department of Agriculture, Agricultural Research Service, Eastern Regional Research Center, Wyndmoor, PA, United States; ^2^Dairy and Functional Foods Research Unit, United States Department of Agriculture, Agricultural Research Service, Eastern Regional Research Center, Wyndmoor, PA, United States; ^3^Core Technologies Unit, United States Department of Agriculture, Agricultural Research Service, Eastern Regional Research Center, Wyndmoor, PA, United States

**Keywords:** *Campylobacter*, transcription, mRNA, regulation, SNP, cysteine

## Abstract

A single nucleotide polymorphism (SNP) in the 126 bp untranslated region (UTR) directly upstream of *Campylobacter jejuni’s cysM* (cysteine synthase) results in significant effects on gene transcription. UTR sequences, containing the predicted promoter region of *cysM*, from 264 different strains were compared, and revealed a SNP twenty nucleotides upstream of the *cysM* translation start site. In 219 strains the UTR sequence contained a guanine at this locus, and the remaining 45 strains had an adenine at the same position. Strains possessing the guanine SNP showed higher amounts of *cysM* transcripts compared to adenine SNP strains. When both UTR regions were cloned upstream of the major flagellar subunit (*flaA*) the guanine SNP UTR resulted in significantly greater levels of *flaA* transcription compared to the adenine SNP containing UTR. Additionally, when the UTR containing the guanine SNP was fused to *flaA*, motility was restored for a *flaAB* null mutant. Motility was not rescued initially when *flaA* was fused to the UTR containing the adenine SNP UTR. However, when the *flaAB* null mutant, containing a copy of *flaA* fused to the adenine-containing UTR, was incubated in Brucella broth for a minimum of two consecutive passages each lasting 48 h, transcription of *flaA* increased and motility was restored. Additional analysis of the *flaA* mRNA produced by the strain containing the adenine SNP UTR fused to *flaA* grown in Brucella broth versus agar suggests that the effects on motility occurred through blocking of full-length mRNA production.

## Introduction

*Campylobacter jejuni* is recognized as the causative agent for the greatest number of bacterial foodborne gastrointestinal disease cases recorded each year in the developed world (Marder et al., 2017; European Food and Safety Authority, 2021). In the United States alone it is estimated that ∼1 million cases of foodborne illness are caused annually by *Campylobacter* at an economic cost of ∼2 billion dollars (Kaakoush et al., 2015). The bacterium is particularly virulent to humans, requiring an infectious dose of as little as 500 organisms (Black et al., 1988). Additionally, *C. jejuni* is able to survive and persist in a diverse collection of environments, including both wild and farmed animals and water sources (Levin, 2007; Gillespie et al., 2002). *Campylobacter jejuni’s* ability to survive, adapt, and infect within diverse environments likely necessitates the organism’s strain to strain genetic variations (Burnham and Hendrixson, 2018).

The advances in the availability and affordability of whole genome sequencing have greatly assisted in the process of identifying the differences between *Campylobacter* genomes. However, identifying genetic differences is not sufficient to understand their effect on pathogenesis, thus it is necessary to fully characterize gene functions and define the roles that the genetic changes have on *Campylobacter* physiology. Problematically, there has been a lack of molecular tools available for laboratory research on *C. jejuni*. In response to this need, a two-plasmid system was developed for the cloning and constitutive expression of exogenous genes inserted within the *C. jejuni* chromosome (Uhlich et al., 2022). Continuing research has focused on the UTR sequence that is utilized to drive constitutive gene transcription in the two-plasmid system. The UTR originates from a sequence found directly upstream of the cysteine synthase gene (*cysM*) within the *Campylobacter* genome, and is responsible for transcription of this gene (Garvis et al., 1997). In *C. jejuni*, *cysM* is needed for production of the amino acid cysteine, which occurs from the transfer of sulfur from environmental sources to serine (Hitchcock et al., 2022). *C. jejuni* require cysteine for growth, therefore *cysM* is essential under nutrient limiting conditions when cysteine cannot be scavenged from the environment (Alazzam et al., 2011). In analyzing the UTR sequence upstream of *cysM*, a well-conserved single nucleotide polymorphism (SNP) in the *C. jejuni* population was identified. The SNP was located near the proposed promoter region of *cysM* between the putative ribosomal binding site and −10 RNA polymerase site (Garvis et al., 1997). The effects, of this SNP in the UTR sequence, were analyzed to determine the influence upon *cysM* transcription.

## Materials and methods

### Bacterial strains and plasmids

All bacterial strains and plasmids used in this study are listed in Table 1. For long term storage, *Campylobacter* strain freezer stocks were maintained in Brucella broth (Becton Dickinson, Sparks, USA) plus 15% glycerol frozen at −80°C. Strain cultures were revived from freezer stocks directly onto Brucella agar (1.5%) plates. Kanamycin (15 μg mL^1^ final concentration) or tetracycline (5 μg mL^1^ final concentration) was added to Brucella agar plates or Brucella broth when growing strains possessing a cloned *aph*(3) or *tetO* gene, respectively. All *C. jejuni* cultures were incubated in a microaerobic (5% O_2_, 10% CO_2_, 85% N_2_) growth chamber (Concept-M, Baker, Sanford, USA) at 42°C.

**TABLE 1:** Strains, plasmids and primers utilized in research.

Strain	Description	Source or references
*C. jejuni* RM1285	Clinical isolate	Gunther et al., 2015
*C. jejuni* RM1188	Environmental isolate	Miller et al., 2005
*C. jejuni* FSIS136	Clinical isolate (FSIS11705136) (210334145)	Food Safety Inspection Service
*C. jejuni* CDC9511	Clinical isolate CDC9511(2012D-9511)	Centers for Disease Control
*C. jejuni* RM3194	Clinical isolate	Gunther et al., 2016
*C. jejuni* RM3194 + express	RM3194 with just the *cysM* UTR (G_20_ variety) expression element integrated into chromosome; Kn^R^	Gunther et al., 2023
*C. jejuni* RM3194Δ*flaAB:tet*	RM3194 with deletion of *flaA* and *flaB*; Tc^R^	Uhlich et al., 2022
*C. jejuni* RM3194Δ*flaAB:tet* + *flaA*comp (G_20_)	RM3194 with deletion of *flaA* and *flaB*; with integrated *cysM* UTR (G_20_ variety)-*flaA* expression element; Tc^R^ and Kn^R^	Uhlich et al., 2022
*C. jejuni* RM3194Δ*flaAB:tet* + *flaA*comp (A_20_)	RM3194 with deletion of *flaA* and *flaB*; with integrated *cysM* UTR (A_20_ variety)-*flaA* expression element; Tc^R^ and Kn^R^	This study
**Plasmid**	**Description**	**Source**
pBlueKan + *cysM*^Pro^ (G_20_)	pBluescriptII KS + with cloned *aph*(3), *cysM* UTR of the G_20_ variety, and unique 3′ *Sma*I and *Xho*I cloning sites; Kn^R^; Ap^R^	Uhlich et al., 2022
pBlueKan + *cysM*^Pro^ (A_20_)	pBluescriptII KS + with cloned *aph*(3), *cysM* UTR of the A_20_ variety, and unique 3′ *Sma*I and *Xho*I cloning sites; Kn^R^; Ap^R^	This study
pCJR01	pBC SK:rRNA with unique *Not*I cloning site; Cm^R^	Uhlich et al., 2022
pBlueKan + *cysM*^Pro^ (A_20_)-*flaA*	pBlueKan + *cysM*^Pro^ (A_20_) with Gibson-cloned *flaA*; Kn^R^; Ap^R^	This study
pCJR01comp (A_20_)-*flaA*	pCJR01 with cloned *aph*(3), *cysM* UTR(A_20_),and *flaA*; Kn^R^; Cm^R^	This study
**Primers**	**Description (5′–3′)**	**Source**
*cysM*3194rev + blueex	ctcgaggatcccgggaattttaatatccttttttatttaataatg	This study
*cysM*for + Kanex	gaggatatcggggaagaacagtatg	This study
Bluefor + *cysM*ex	gatcctcgaggcggccgcggtacccag	This study
Kanrev + *cysM*ex	gatatcctccctgatcgaccg	This study
*FlaA*F + *cysM*PRex	aaggatattaaaattatgggatttcgtattaacaccaatg	This study
3194*cysM*PR + *FlaA*Fex	aatacgaaatcccataattttaatatccttttttatttaataatgatagttttataaaag	This study
3194pBlue + *Fla*REx2	gtttacaaaagctggggatcctcgaggcggccgc	This study
3194*FlaA*R + pBlueEx2	cggccgcctcgaggatccccagcttttgtaaactactgtagt	This study
*rpoA*2B_F	tcttcaagcataccacgcat	This study
*rpoA*2B_R	atcaccctagcccatccttt	This study
*cysM*2_F	tagctggttttggaactggc	This study
*cysM*2_R	tagaggctcgactccaacaa	This study
*flaA*2D_F	aggctatggatgagcaactt	This study
*flaA*2D_R	actttgtccatcttgagccg	This study
*flaA*_cDNA	agagccactttgagccaaga	This study
*flaA*2Dshort_F	tttcaaatcggcgcaagttc	This study
*flaA*2Dmedium_F	catcggtggtggagctttta	This study
*flaA*2Dlong_F	tgcaggttcgggttattctg	This study
*flaA*2B_R	gaaccaatgtcggctctgat	This study

### Bioinformatic analysis of UTR upstream of *cysM*

Blast analysis of the UTR directly upstream of *cysM* was performed as follows: the NCBI genome website was searched using “*Campylobacter jejuni* subsp. *jejuni* AND (latest OR ‘latest genbank’) AND all NOT anomalous” search words on 05-12-2023 (Altschul et al., 1990). This downloaded 326 genome fasta sequences. A 126-bp UTR sequence was used against the downloaded 326-genome sequences using Blastn + software using default parameters. The subject sequences were selected for 126 bp length and no-gaps in sequences. This produced 264 sequences of 126 bases long. The 264 sequences were aligned with MAAFT software (v7.487) with FFT-NS-i parameter (Katoh and Standley, 2013). The UTR sequence was analyzed for regulatory elements and transcription factor binding sites using bprom software (Solovyev and Salamov, 2011).^1^

### Construction of A_20_ *cysM* UTR-*fla*A strain

The *C. jejuni* strain RM3194Δ*flaAB:tet* + *flaA*comp (G_20_) had been previously constructed (Uhlich et al., 2022). In order to investigate the differences in transcription when an adenine was at the 20th position in the UTR compare to guanine we need to construct a strain the same as RM3194Δ*flaAB:tet* + *flaA*comp (G_20_), but with the desired single nucleotide change. Plasmid pBlueKan + *cysM^Pro^* (G_20_) carrying a guanine at the 20th site in the UTR was PCR amplified using two sets of primers in order to achieve a Gibson style assembly. Primers *cysM*3194rev + blueex and *cysM*for + Kanex were used to amplify the *cysM* UTR and replace the guanine nucleotide with an adenine. Primers Bluefor + *cysM*ex and Kanrev + *cysM*ex were used to amplify the vector backbone. Using the NEBuilder HiFI DNA Assembly kit (NEB, Ipswitch, MA, USA) and the overlap extensions built into the PCR primers the plasmid pBlueKan + *cysM^Pro^* (G_20_) was reconstructed as plasmid pBlueKan + *cysM^Pro^* (A_20_) with the adenine in the desired location. Next *flaA* from strain RM3194 was cloned into the newly constructed plasmid pBlueKan + *cysM^Pro^* (A_20_), again using a Gibson style assembly. Primers 3194pBlue + *FlaA*REx2 and 3194*FlaA*R + pBlueEx2 were used to clone *flaA* from RM3194 and primers *FlaA*F + *cysM*PRex and 3194*cysM*PR + *Fla*AFex were used to amplify the pBlueKan + *cysM^Pro^* (A_20_) plasmid. Again using the NEBuilder kit and the overlap extensions built into the primer sets, *flaA* was cloned into plasmid pBlueKan + *cysM^Pro^* (A_20_) producing the new plasmid, pBlueKan + *cysM^Pro^* (A_20_)-*flaA*. The pBlueKan + *cysM^Pro^* (A_20_)-*flaA* plasmid was digested with *Not*I (NEB) and the fragment containing the kanamycin resistance gene, the A_20_ *cysM* UTR and *flaA* was gel purified away from the plasmid backbone. The purified fragment was ligated with the pCJR01 vector that was also digested with *Not*I. The resulting plasmid was designated, pCJR01comp (A_20_)-*flaA* and subsequently electroporated into strain *C. jejuni* RM3194Δ*flaAB:tet* (Davis et al., 2008). Before electroporation the plasmid pCJR01comp (A_20_)-*flaA* was sequenced to ensure that no additional changes to the *cysM* UTR or *flaA* had occurred during the cloning process. Transformants were selected for on Brucella agar plus kanamycin and cells from the resulting colonies were transferred individually onto Brucella agar plus chloramphenicol and Brucella agar plus kanamycin plates. Successful double crossover integration events of the *cysM* UTR *flaA* into the bacteria chromosome were identified by the presence of kanamycin resistance and the absence of chloramphenicol resistance. All successful transformants were directly checked for motility on low agar Brucella plates, no transformants were motile. Several colonies were then chosen at random, stored as freezer stocks and designated, as RM3194Δ*flaAB:tet* + *flaA*comp (A_20_) strains.

### Growth conditions to produce different expression profiles in the A_20_ *cysM* UTR-*flaA* strain

Strains RM3194Δ*flaAB:tet* + *flaA*comp (G_20_) and RM3194Δ*flaAB:tet* + *flaA*comp (A_20_) were taken directly from freezer stocks and grown on Brucella agar (1.5%) plus Kanamycin. When either strain was grown (for 16 to 48 h) on solid agar media directly from freezer stocks or passed from one Brucella agar plate to the next, RM3194Δ*flaAB:tet* + *flaA*comp (G_20_) was referred to as a G_20_ solid media culture (GSM) and RM3194Δ*flaAB:tet* + *flaA*comp (A_20_) was referred to as an A_20_ solid media culture (ASM). Cells from either of these strains taken from agar plates and inoculated into Brucella broth (with kanamycin) and grown for up to 48 h were still classified as GSM or ASM strains since no changes in the motility of the ASM strain was observed at this point. However, when cells were taken from the liquid cultures and placed into fresh Brucella broth plus kanamycin and incubated again for at least 48 h, changes in the motility of the formally non-motile ASM strain began to be observed. Therefore, these cultures were renamed as G_20_ liquid media cultures (GLM) for RM3194Δ*flaAB:tet* + *flaA*comp (G_20_) and A_20_ liquid media cultures (ALM) for RM3194Δ*flaAB:tet* + *flaA*comp (A_20_). Cells from GLM and ALM cultures after 48 h of growth were routinely transferred into fresh Brucella broth plus kanamycin to maintain the health of the bacterial cultures.

### *C. jejuni* motility assay

Low concentration agar (0.3%) Brucella plates were used to measure the motility of the *C. jejuni* strains in this study, with kanamycin or tetracycline being added to the plates where necessary (Barrero-Tobon and Hendrixson, 2012). Bacterial strains to be evaluated were initially grown overnight in 3 mL of Brucella media plus kanamycin or tetracycline if appropriate. The following day 1 μL drops of the individual *C. jejuni* cultures were injected into the center of the appropriate 0.3% agar Brucella plates. Plates were incubated for 16 h in a microaerobic atmosphere chamber at 42°C. Following incubation, the diameters of the resulting circular bacterial migration zones were measured to the closest millimeter. The average motility for individual strains were then calculated from at least three experimental replicates.

### Electron microscopy

Fifty microliters of the appropriate *C. jejuni* strain, were placed on acetone cleaned 12 mm Micro-cover glass slides (Thermo Scientific Portsmouth, NH, USA) and allowed to adhere for 30 min. Samples were then covered with 2 ml of 2.5% glutaraldehyde, (Electron Microscopy Sciences, Hatfield, PA, USA) and were allowed to fix for 30 min. The samples were then rinsed twice for 30 min each with 2–3 mL of the 0.1 M imidazole, (Electron Microscopy Sciences, Hatfield, PA, USA), followed by 30 min intervals each of 50, 80, 90% ethanol (The Warner-Graham Company, Cockeysville, MD, USA), 2–3 ml each. The samples were then washed and held three times with 2 mL of 100% ethanol before being critically point dried. The samples were stacked in a wire basket, separated by cloth, and placed in a Critical Point Drying Apparatus, (Denton Vacuum, Inc., Cherry Hill, NJ, USA), using liquid carbon dioxide (Welco Co, Allentown, PA, USA) for approximately 20 min. The samples were mounted on stubs and sputter gold coated for 1 min (EMS 150R ES, EM Sciences, Hatfield, PA, USA). Samples were then viewed with a FEI Quanta 200 F Scanning Electron Microscope, (Hillsboro, OR, USA) with an accelerating voltage of 10KV in high vacuum mode.

### Transcription analysis of *flaA* and *cysM*

RNA was isolated from 2 mL aliquots of each of the designated *C. jejuni* strains incubated in Brucella broth supplemented with kanamycin or tetracycline where appropriate. Samples were collected for RNA isolation once they had reached optical densities of between 0.1 and 0.3 when measured at 600 nm (OD_600_) using a Genesys 30 spectrometer (ThermoFisher Scientific). Cells were collected by centrifugation (7,000 × *g*) and RNA was isolated using the Invitrogen PureLink RNA Kit (ThermoFisher Scientific). The RNA samples were next DNase I treated with Invitrogen’s Turbo Dnase kit (ThermoFisher Scientific). Finally, cDNA synthesis was accomplished using Invitrogen’s SuperScript III First-Strand Synthesis Kit (ThermoFisher Scientific) and a Bio-Rad T100 thermocycler (Bio-Rad, Hercules, CA, USA) following manufacturer’s instructions.

Primer sets used for the quantitative PCR reactions were as follows: *rpoA*2B_F (forward primer) and *rpoA*2B_R–(reverse primer) were used to amplify the *rpoA* housekeeping gene transcription, which served as an internal control to normalize the different starting RNA levels between the samples. *cysM*2_F and *cysM*2_R were used to measure the native *cysM* transcription. *flaA*2D_F and *flaA*2D_R were used to measure transcription of the cloned *flaA*. RT-PCR reactions were performed in 8 tube strips using a Roche LightCycler 96 system (Roche Scientific). Reactions were 20 μL in total volume including: 10 μL of FastStart Essential DNA Green Master Mix (Roche), 1 μL of each 10 μM primer forward and reverse, and 8 μL of cDNA previously diluted 1:40. The amplification protocol was as follows: initial denaturation step at 95°C for 10 min, followed by 45 cycles of 95°C for 10 s, 58°C for 10 s, and 72°C for 10 s and concluded with a melting curve analysis. The LightCycler 96 system software was used to assess the PCR results. Each set of RT-PCR experiments also included “no reverse transcriptase” samples to control for genomic DNA contamination as well as “no template” controls lacking only the cDNA template. For each sample the relative transcription level of the target gene (*cysM* or *flaA*) was calculated as a ratio to the housekeeping gene (*rpoA*) to allow for comparisons between strains and conditions. Equation: ratio = E_*R*_*^Cq^*_*R*_/E_*T*_*^Cq^*_*T*_ (E_*R*_, quantification efficiency of reference gene, E_*T*_, quantification efficiency of target gene, Cq_*R*_, quantification cycle of reference, Cq_*T*_, quantification cycle of target).

### Analysis of mRNA transcript lengths from ALM and ASM samples

RNA was isolated from ASM and ALM samples as described above. The resulting RNA concentrations from each sample were measured using a NanoDrop spectrophotometer (ThermoFisher Scientific) and the RNA was then diluted to produce equal concentrations of starting RNA. cDNA was produced from RNA in the same manner as before except a single primer *flaA*_cDNA, specific to *flaA* mRNA was used to produce cDNA only from *flaA* mRNA in the isolated RNA samples. The *flaA* specific primer (*flaA*_cDNA) sequence localizes to the 3′ end of the theoretical *flaA* mRNA. Next the *flaA* specific cDNA produced in the previous step were used as template in PCR amplification using the following four forward primers *flaA*2D_F, *flaA*2Dshort_F, *flaA*2Dmedium_F and *flaA*2Dlong_F each localizing at increasing distance from the 5′ of the *flaA* cDNA and paired separately with the reverse primer, *flaA*2B_R. PCR conditions were: 95°C for 10 min followed by 35 cycles of 95°C for 30 s, 57°C for 30 s, 72°C for 90 s with a final single 72°C for 10 min. PCR amplification used the Qiagen Multiplex PCR kit following manufacturer’s recommended method. Subsequent PCR products were visualized on a 1% DNA gel subsequently stained with GelRed (Thomas Scientific).

### Statistical analysis

At least three separate experimental replicates were used to produce all mean and standard error values reported in this paper. The one-way analysis of variance (ANOVA) test was used to determine if mean values compared in this study were significantly different. Subsequent to the ANOVA analysis, the Tukey HSD test was used to separate the mean values. Significant differences were defined as *P*-values ≤ 0.05.

## Results

### Sequence analysis of UTR directly upstream of *cysM*

**FIGURE 1:**
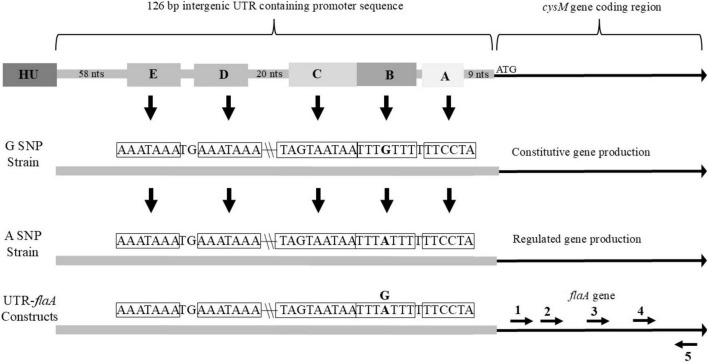
Overview of 126 bp intergenic UTR found upstream of *cysM* in nature, that was cloned in front of *flaA* for this study. Important sequence features and a single nucleotide polymorphism (SNP) for strains belonging to the G-SNP or A-SNP groups and their resulting differences in downstream gene production are also noted. (A) predicted ribosome binding site (sequence within corresponding box indicated by arrow); (B) SNP site with surrounding sequence complementary to upstream sequences; (C) predicted fur binding site; (D) first sequence complimentary to sequence B; (E) second sequence complimentary to sequence B. Relative locations of primers (1) *flaA*2D_F, (2) *flaA*2Dshort_F, (3) *flaA*2Dmedium_F, (4) *flaA*2Dlong_F and (5) *flaA*2B_R within the *flaA* sequence of the UTR-*flaA* constructs. Location of HU gene directly upstream of UTR included in figure.

The 126 bp UTR between the cysteine synthase (*cysM*) gene and DNA-binding protein HU gene is well conserved between sequenced *C. jejuni* strains. In this study 264 sequences of this region were aligned and observed for sequence variations. Within the 264 sequences only eighteen nucleotide sites were observed to vary over the 126 bp region. Additionally, most of these nucleotide substitutions were rare with any of the nucleotide variations only occurring in a few strains in total. However, at one site a nucleotide substitution was observed in a sizable percentage of the 264 sequences, with 219 sequences having a guanine (G) nucleotide at this location and the remaining 45 sequences alternately having an adenine (A). Nucleotides within the 126 bp region were numbered starting from the nucleotide directly upstream of the first nucleotide of the *cysM* coding sequence (#1) and ended at the nucleotide immediately downstream of the last nucleotide of the HU DNA-binding gene (#126). The predominant SNP (G/A) was located at position number 20 with the SNPs designated as G_20_ or A_20_. Further analysis of the sequences identified a putative ribosomal binding site ATCCTT running from nucleotides 10 to 15 of the UTR (Figure 1). Additionally, a possible fur binding site AATAATGAT (nucleotides 24 to 32) and an integration host factor (IHF) binding site, which contained the G_20_/A_20_ SNP [TTTTA(G)TTT, nucleotides 16 to 23] were identified. While it is believed that *C. jejuni* lack most transcription factors, including IHF, it does possess the gene for HU which appears to perform many of the same functions of IHF (Stojkova et al., 2019).

### *cysM* transcription levels are higher in *C. jejuni* strains with the G_20_ SNP compared to A_20_ possessing strains

**FIGURE 2:**
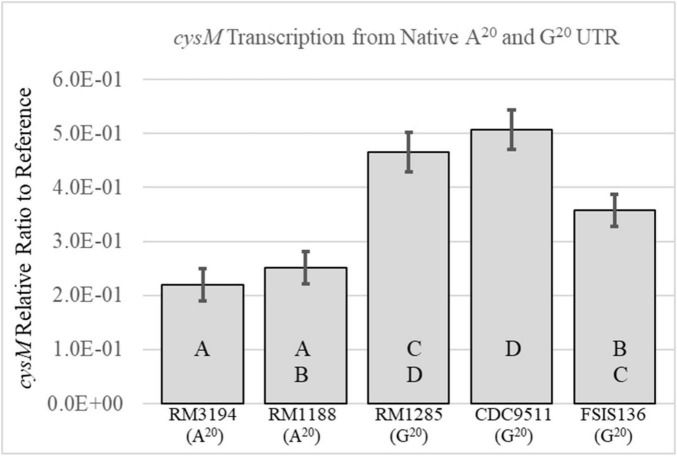
Comparison of the *cysM* transcription levels between strains with an A_20_ UTR upstream of *cysM*—RM3194, RM1188 or a G_20_ UTR upstream of *cysM*—RM1285, CDC9511, FSIS136. Significantly different transcription values are indicated by different letter notations.

Sequenced strains with the G_20_ or A_20_ SNP in the UTR upstream of *cysM* were selected from the laboratory strain collection for transcription analysis. The strains were grown in Brucella broth and collected during the exponential phase of growth, at OD_600_ values of between 0.1 and 0.3. Total RNA was isolated and cDNA produced from the mRNA transcripts. Quantitative RT-PCR was used to measure *cysM* transcription levels for each strain (Figure 2). The values for the *cysM* transcription levels were presented as ratios to the transcription levels of the housekeeping gene, *rpoA*, allowing for normalization of concentration differences between various samples. On average the G_20_ SNP strains (FSIS136, CDC9511, and RM1285) had higher relative transcription levels of *cysM* when compared to strains with the A_20_ SNP (RM3194, RM1188). The average transcription levels of the A_20_ SNP strains RM3194 and RM1188 did not significantly differ from one another with values of 0.22 and 0.25, respectively. Within the G_20_ SNP strains, there was variation in the *cysM* transcription levels. Strain FSIS136 (0.38) differed significantly from CDC9511 (0.51) with regards to *cysM* transcription, while strain RM1285 (0.47) did not differ significantly from CDC9511 or FSIS136. These results suggested that during exponential growth in a rich medium, the different SNPs contributed to differences in the regulation of *cysM* transcription.

### The UTRs, containing the A_20_ or G_20_ SNPs, when used to drive *flaA* transcription in a *flaAB* deletion strain produced significantly different motility profiles

**FIGURE 3:**
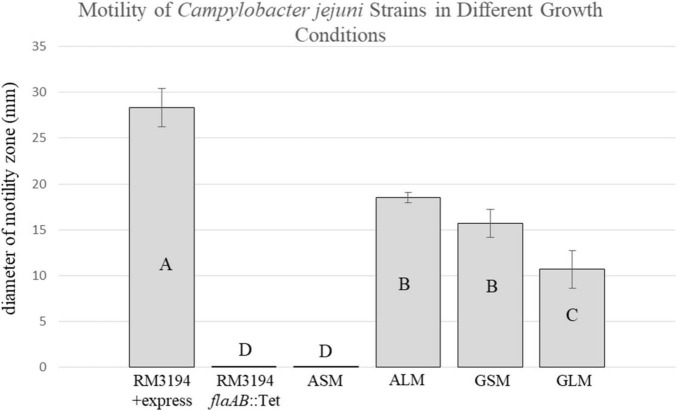
Motility of *C. jejuni* strains. Strain positive for motility: RM3194 + express. Strain negative for motility: RM3194Δ*flaAB:tet.* Strains unknown for motility: RM3194Δ*flaAB:tet* + *flaA*comp (A_20_) grown on solid media (ASM), RM3194Δ*flaAB:tet* + *flaA*comp (A_20_) grown in liquid media (ALM), RM3194Δ*flaAB:tet* + *flaA*comp (G_20_) grown on solid media (GSM), RM3194Δ*flaAB:tet* + *flaA*comp (G_20_) grown in liquid media (GLM). Significantly different motility values are indicated by different letter notations.

The UTR upstream of *cysM* is presumed to contain the promoter region for the gene. The G_20_ SNP version of the UTR was previously used in research studies as a constitutive promoter to drive expression of exogenous genes cloned into *C. jejuni* (Uhlich et al., 2022; Gunther et al., 2023). In order to determine how the A_20_ SNP might affect the transcription of recombinant genes cloned within the *C. jejuni* chromosome, the *cysM* UTR contained within of the cloning vector pBlueKan + *cysM^Pro^* (G_20_) was changed to adenine (A_20_). This allowed for the construction of *C. jejuni* strain RM3194Δ*flaAB:tet* + *flaA*comp (A_20_). *C. jejuni* RM3194Δ*flaAB:tet* is a clinical strain with both flagellar structural genes *flaA* and *flaB* removed. The exogenous *flaA* cloned behind an A_20_ UTR was inserted within the *C. jejuni* RM3194Δ*flaAB:tet* chromosome between the 16S and 23S ribosomal RNA genes. The resulting strain, RM3194Δ*flaAB:tet* + *flaA*comp (A_20_), was identical to the previously constructed strain, *C. jejuni* RM3194Δ*flaAB:tet* + *flaA*comp (G_20_) (Uhlich et al., 2022), with the exception of the A_20_ SNP modification now upstream of the cloned flaA gene. The *C. jejuni* RM3194Δ*flaAB:tet* + *flaA*comp (A_20_) strain was originally believed to be non-motile, however, it was observed to become motile when incubated in Brucella broth for two or more consecutive cultures each lasting 48 h. To illustrate the motility differences produced by the two UTR SNP forms, the strains *C. jejuni* RM3194Δ*flaAB:tet* + *flaA*comp (A_20_) and *C. jejuni* RM3194Δ*flaAB:tet* + *flaA*comp (G_20_) were grown either on solid media (1.5% agar) to form cultures designated GSM (G_20_ UTR) and ASM (A_20_ UTR) or within liquid medium with at least two passages into fresh medium resulting in cultures designated GLM (G_20_ UTR) and ALM (A_20_ UTR). The motilities of each of these four cultures, were compared on low percentage agar, along with a positive control strain, *C. jejuni* RM3194 + express, and a negative control strain, *C. jejuni* RM3194Δ*flaAB:tet* (Figure 3). On average the positive control strain with the wild-type copies of both flagella structural genes, driven by their native promoters, produced motility zones of 28.3 mm. The GSM and GLM cultures produced average motility zones of 15.7 and 10.6 mm, respectively, with the different growth conditions having only a small influence on cell motility. This differed significantly from the influence that the growth conditions had on motility when *flaA* was combined with the A_20_ UTR. The ALM culture produced an average motility zone of 18.5 mm while the ASM culture showed no motility appearing identical to the negative control strain that lacked any structural flagella genes. Additionally, cells from the ALM culture when placed onto Brucella agar plates and transferred after 24 h of growth onto fresh agar plates twice more, were observed to completely lose their motility. PCR amplification of the UTR locus from both the ASM and ALM cultures confirmed that the adenine SNP was not modified when switching between motile and non-motile states (data not shown). Additionally, since strain RM3194Δ*flaAB:tet* + *flaA*comp (A_20_) was shown to switch between a motile and non-motile phenotype depending on how it was sub-cultured, suggests that a mutational event in a location other than the UTR is not responsible for the observed changes.

### Electron microscopy confirms that the presence or absence of flagella on the surface of the *C. jejuni* strain correlates with the differences observed in motility of the A_20_ and G_20_ UTR plus *flaA* strains

**FIGURE 4:**
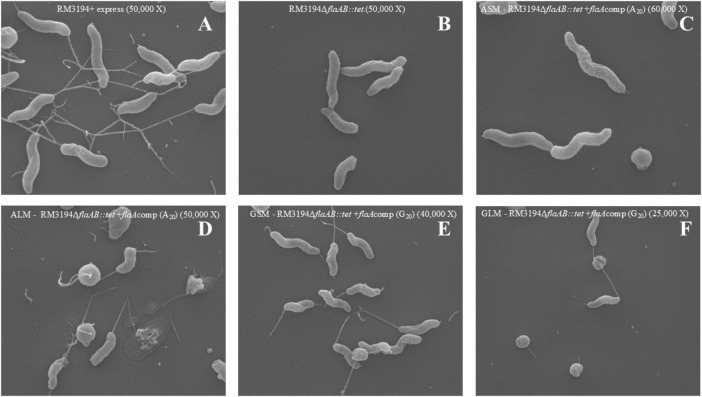
Scanning Electron Microscopy of G_20_ or A_20_ UTR plus *flaA* strains grown to produce different motility. **(A)** Positive control, RM3194 + express (50,000 X). **(B)** Negative control, RM3194Δ*flaAB:tet* (50,000 X). **(C)** Non-motile strain, ASM–RM3194Δ*flaAB:tet* + *flaA*comp (A_20_) (60,000 X). **(D)** Motile strain, ALM–RM3194Δ*flaAB:tet* + *flaA*comp (A_20_) (50,000 X). **(E)** Motile strain GSM–RM3194Δ*flaAB:tet* + *flaA*comp (G_20_) (40,000 X). **(F)** Motile strain, GLM–RM3194Δ*flaAB:tet* + *flaA*comp (G_20_) (25,000 X).

The strains and cultures used in the previous motility experiments (Figure 3) were assessed by scanning electron microscopy to identify differences in cell structure that might affect motility. Representative images for each culture condition are presented in Figure 4. Flagella are readily visible on the cells comprising the positive control strain (Figure 4A). Conversely, no flagella were visible on cells of the negative control strain (Figure 4B). The non-motile ASM culture (Figure 4C) appears similar to the negative control without any flagella readily visible on the cells surface. The motile ALM (Figure 4D), GSM (Figure 4E), and GLM (Figure 4F) each have flagella visible on the cells within the respective cultures.

### Transcription levels of *flaA* in the RM3194Δ*flaAB:tet* + *flaA*comp (A_20_) strain differ between the motile ALM cultures and the non-motile ASM cultures

**FIGURE 5:**
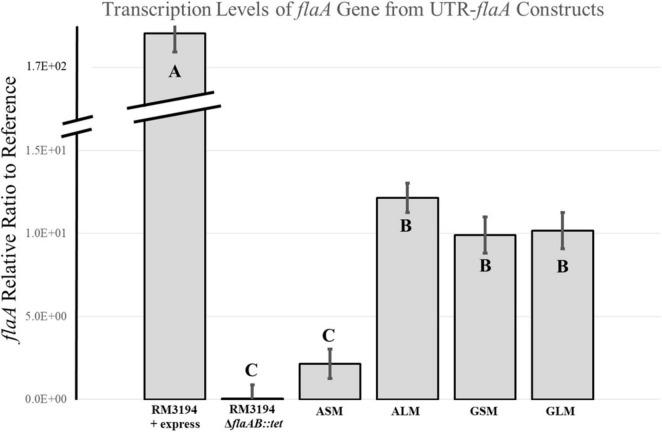
Transcription analysis of *flaA* downstream of G_20_ or A_20_ UTR sequence strains, RM3194Δ*flaAB*:*tet* + *flaA*comp (G_20_) or RM3194Δ*flaAB*:*tet* + *flaA*comp (A_20_), grown to produce different motility. Results are presented as a relative ratio of *flaA* transcription to the housekeeping gene *rpoA* transcription to control for sample-to-sample variations. Significantly different transcription values are indicated by different letter notations.

Quantitative RT-PCR was used to determine the *flaA* transcription levels in the positive control, RM3194 + express, and negative control strain, RM3194Δ*flaAB:tet*; as well as strain, RM3194Δ*flaAB:tet* + *flaA*comp (A_20_), grown under conditions producing motility and observable flagella (ALM) and the conditions without motility or observable flagella (ASM). The transcription of *flaA* was also measured for the strain, RM3194Δ*flaAB:tet* + *flaA*comp (G_20_), grown under GLM and GSM conditions, both of which previously produce motility and observable flagella. Results for these experiments are presented in Figure 5. Not surprisingly strain RM3194 + express had the highest level of *flaA* transcription since it also previously had demonstrated the greatest motility. The non-motile negative control strain with no *flaA* or *flaB* predictably did not show any measurable *flaA* transcription. The strain and culture conditions that previously demonstrated motility and flagellar expression, ALM, GSM, and GLM all had similar average levels of *flaA* transcription. Unsurprisingly, these levels of *flaA* transcription were significantly less than that in the positive control strain given the superior motility demonstrated by the control strain. The non-motile, no observable flagella culture, ASM, had significantly lower *flaA* transcription levels when compared to ALM, GSM and GLM cultures. In fact the *flaA* transcription of RM3194Δ*flaAB:tet* + *flaA*comp (A_20_) grown in the ASM conditions was not significantly different from the negative control strain which corresponded nicely with the observation that both the negative control and ASM samples were non-motile.

### The ALM growth condition produces increased native *cysM* transcription compared to the ASM growth condition

**FIGURE 6:**
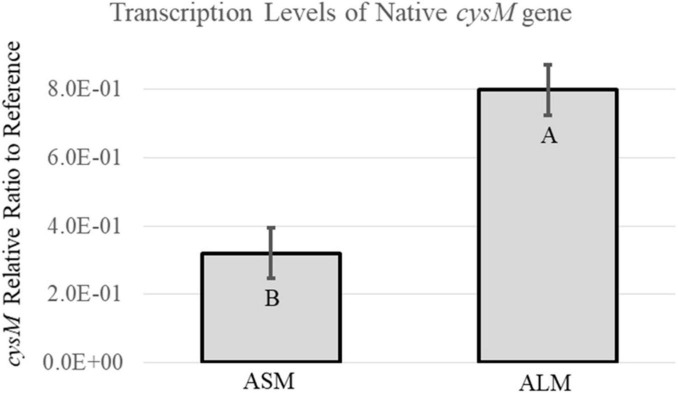
Transcription analysis of native *cysM* in the A_20_ UTR sequence strain, RM3194Δ*flaAB*:*tet* + *flaA*comp (A_20_), grown to produce different motility. Results are presented as a relative ratio of *cysM* transcription to the housekeeping gene *rpoA* transcription to control for sample-to-sample variations. Significantly different transcription values are indicated by different letter notations.

Strain RM3194Δ*flaAB:tet* + *flaA*comp (A_20_) grown in ALM or ASM conditions demonstrated differences in motility, observed flagella and *flaA* transcription levels. Since the parent strain, RM3194, is known to possess an A_20_ UTR upstream of *cysM*, quantitative RT-PCR experiments were performed to determine how the ALM and ASM growth conditions affected the transcription of the native *cysM*. The results of these experiments are presented in Figure 6. The ALM condition, on average, resulted in significantly greater levels of *cysM* transcription compared to the ASM conditions, which agreed with previous results measuring the expression of recombinant *flaA* in strain RM3194Δ*flaAB:tet* + *flaA*comp (A_20_) (Figure 5). This result suggests that transcription of any gene cloned downstream of the A_20_ UTR will be regulated differently depending on the growth conditions under which the host strain is cultured.

### ALM and ASM growth conditions result in different *flaA* cDNA lengths produced from strain RM3194Δ*flaAB:tet* + *flaA*comp (A_20_) RNA

**FIGURE 7:**
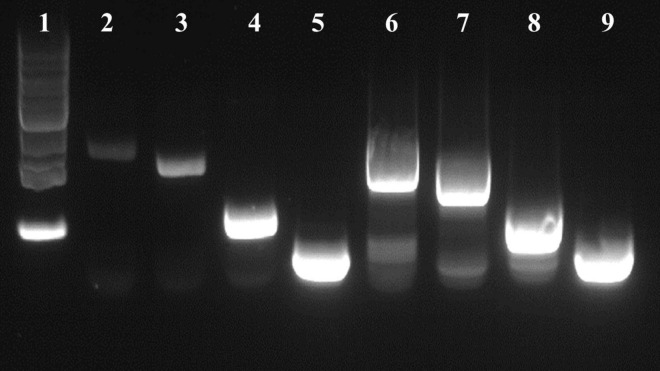
DNA gel visualization of PCR products from amplifications of *flaA* cDNA produced from strain RM3194Δ*flaAB:tet* + *flaA*comp (A_20_) grown in ASM or ALM conditions, using the following primer combinations. Lane (1) 1kb DNA Ladder (Invitrogen), lane (2) ASM with *flaA*2D_F/*flaA*2B_R, lane (3) ASM with *flaA*2Dshort_F/*flaA*2B_R, lane (4) ASM with *flaA*2Dmedium_F/*flaA*2B_R, lane (5) ASM with *flaA*2Dlong_F/*flaA*2B_R, lane (6) ALM with *flaA*2D_F/*flaA*2B_R, lane (7) ALM with *flaA*2Dshort_F/*flaA*2B_R, lane (8) ALM with *flaA*2Dmedium_F/*flaA*2B_R, lane (9) ALM with *flaA*2Dlong_F/*flaA*2B_R.

A *flaA* specific primer was used to only produce *flaA* cDNA from RNA collected when strain RM3194Δ*flaAB:tet* + *flaA*comp (A_20_) was grown in ALM or ASM conditions. The total RNA amounts present in the ALM and ASM samples were selectively diluted to equalize the starting RNA concentrations between the samples. The resulting *flaA* cDNAs isolated from the two different growth conditions were then used as template for PCR amplification. The forward primers used for the PCR amplifications were designed to bind to the *flaA* cDNA sequences at increasing distances from the 5′ end of the gene. Primer *flaA*2D_F binds to the sequence starting 239 bps downstream of the ATG start codon of *flaA*; while *flaA*2Dshort_F binds 438 bps downstream of the ATG start codon, *flaA*2Dmedium_F binds 935 bps and *flaA*2Dlong_F binds 1,223 bps downstream of the gene’s ATG start codon. All forward primers were paired with the same reverse primer *flaA*2B_R. Therefore, when paired with *flaA*2B_R for PCR amplification *flaA*2D_F produces a 1285 bp fragment, *flaA*2Dshort_F produces a 1,085 bp fragment, *flaA*2Dmedium_F produces a 588 bp fragment and *flaA*2Dlong_F produces a 300 bp fragment. The relative location of the primers within *flaA* are presented in Figure 1. When the resulting PCR amplifications were visualized (Figure 7) only extremely faint bands were visible in lanes 2 and 3, which are the results of amplifying the cDNA from the ASM sample using forward primers *flaA*2D_F and *flaA*2Dshort_F, respectively. However, in lanes 4 and 5 the forward primers *flaA*2Dmedium_F and *flaA*2Dlong_F produced easily visualized bands amplifying the same ASM sample. With the ALM sample, primers *flaA*2D_F, *flaA*2Dshort_F, *flaA*2Dmedium_F and *flaA*2Dlong_F (lanes 6–9) all produced easily visualized bands. These results suggest that there were significantly fewer full length *flaA* cDNAs produced from the ASM growth samples compared to the ALM samples. This would likely result from there being significantly fewer full length *flaA* mRNA transcripts originally in the ASM samples compared to the ALM samples. The cDNAs, produced from *flaA* mRNA, appear to exist in similar amounts in the ASM (lanes 4 and 5) and ALM (lanes 8 and 9) samples when the PCR reactions amplify the 3′ region of *flaA* cDNA present in the samples. Conversely, it appears for the most part that only the 5′ portions of the *flaA* mRNA sequences from the ALM sample are present and accessible to reverse transcriptase allowing for production of the full length *flaA* cDNA as demonstrated by the larger PCR products manufactured from the PCR reactions from ALM (lanes 6 and 7) but not ASM (lanes 2 and 3) samples. This suggests that the 5′ region of the *flaA* mRNA transcripts present in the ASM sample are missing or in some way not accessible to the reverse transcriptase that would normally produce full length *flaA* cDNA. This same type of experiment was repeated using primers specific for native *cysM*. Similarly, we observed that the 5′ portions of *cysM* mRNA sequences from the ALM samples appeared to be more accessible to reverse transcriptase; allowing for the production of more full length *cysM* cDNA compared to the samples from the ASM conditions. It should be noted that differences observed in full length *cysM* cDNA between ALM and ASM conditions were not as dramatic as that observed with *flaA* cDNA (data not shown).

## Discussion

The observation and subsequent analysis of the SNP that alters gene promoter activity of the UTR upstream of *cysM* produces two different areas for consideration. The first is the evolutionary utility of the existence of two different approaches to regulating *cysM* production in *C. jejuni*. The second is the practical utility of a gene promoting sequence that can be switched between active and inactive states, in terms of its use as a molecular tool for studying gene expression in *C. jejuni*.

The cysteine synthase gene (*cysM*) is required for *C. jejuni’s* production of the amino acid cysteine when it cannot be collected from the bacterium’s surrounding environment (Garvis et al., 1997; Hitchcock et al., 2022). The function of *cysM* is to facilitate the transfer of sulfur to O-acetyl-L-serine to produce cysteine. The sulfur in this process can be derived from hydrogen sulfide which is produced by sulfate reducing bacteria in the human gut (Dordevic et al., 2021). Those same gut bacteria often utilize cysteine in their production of hydrogen sulfide or other metabolites (Braccia et al., 2021; Louis and Flint, 2017). Additionally, a significant portion of the normal human intestinal flora are cysteine auxotrophs and must utilize available cysteine to survive (Starke et al., 2023). Therefore, a *C. jejuni* strain infecting the human gut would likely be in a highly competitive, nutrient limited environment and would likely have to scavenge cysteine or a sulfur source in order to survive (Vorwerk et al., 2014; Stahl et al., 2012). It is therefore possible to think of *cysM* as a virulence factor within the human gut. The gene is necessary for virulence if the organism cannot produce the cysteine it needs to survive from environmental sulfur sources, since it would die and be unable to cause disease (Shayya et al., 2023).

In the presented experiments, *C. jejuni* with the G_20_ SNP containing UTR was observed to produce similar levels of downstream gene transcripts under the two different growth conditions tested (ASM and ALM). Therefore, the G_20_ SNP containing strains should already be producing some level of cysteine synthase when it encounters an environment lacking available cysteine. Conversely, those same G_20_ SNP containing UTR strains would be theoretically wasting valuable resources to always make cysteine synthase even when the *C. jejuni* strain finds itself in an environment abundant in cysteine where production of the synthase would be unnecessary (Anand et al., 2020; Geisel, 2011). In the case of an A_20_ SNP UTR strain, it initially appeared to produce downstream gene transcripts at levels significantly less than the G_20_ SNP strain. However, after incubation in at least two consecutive cultures each lasting 48 h, the A_20_ SNP strains showed a significant increase in the transcription of *cysM*. The potential of the A_20_ SNP UTR to regulate gene transcription could allow those strains with the A_20_ SNP to only produce cysteine synthase when needed. This would help solve any potential issue with wasting energy through constitutive transcription of the synthase gene, as appears could be the case with the G_20_ SNP strain. However, in a direct competition for limited sulfur in the absence of cysteine, the G_20_ SNP strains, which already produce cysteine synthase, might be able to outcompete A_20_ SNP strains, due to the time required for these strains to induce their synthase production. Conversely, in an environment rich in available cysteine the A_20_ SNP stains may be able to conserve energy for growth by not increasing transcription of the cysteine synthase, an option that the G_20_ strains do not possess, potentially leading to the A_20_ strains outcompeting the G_20_ strains.

The exact mechanism utilized by the A_20_ SNP stains to regulate *cysM* transcription is currently unknown. Obviously, the mechanism depends to some degree on the UTR SNP (G_20_ or A_20_) which is the focus of this study. Based on the results presented in Figure 7, it appears that transcriptional differences observed for strains carrying the A_20_ SNP, following exposure to different growth conditions (ASM and ALM), may be due to the use of alternate transcription start sites, which lead to differences in the length of transcripts produced. In the ALM condition mRNA transcripts covered the entire gene length while growth in the ASM condition produced truncated mRNA transcripts covering a fraction of the gene’s coding region. The mechanism therefore could involve the blocking of RNA polymerase from starting mRNA transcription at the proper transcription start site within the UTR upstream of the gene, resulting in the polymerase starting mRNA transcription at alternative sites internal to the gene. It is possible that the SNP change from adenine to guanine could alter the binding of a cofactor which could ultimately prevent proper RNA polymerase binding. This proposed mechanism could also depend on interaction with a putative fur binding site (sequence C, Figure 1) almost directly adjacent to the SNP site (Grabowska et al., 2011; Holmes et al., 2005). Previous research has described single nucleotide changes in intergenic regions that altered the binding of regulatory factors and affected transcription (Cagliero et al., 2007; Pérez-Boto et al., 2015; Zeng et al., 2014). These single nucleotide changes most often blocked binding of repressor elements or restored RNA polymerase recognition sites and in both cases led to constitutive gene transcription. Other research described a change in the regulatory region of a gene that altered the gene’s transcription going from inducible to constitutive, but this change required large sequence deletions or additions and not a SNP (Deng et al., 2015). Alternately, there could be a hairpin structure formed through the binding of the UTR sequence B (Figure 1), which contains the SNP, to the complimentary upstream sequences D or E. This could again block binding of the RNA polymerase to the proper transcription start site. The SNP change from adenine to guanine could be sufficient to reduce the potential hairpin formed by these sequences, thus allowing for the observed constitutive transcription.

While the exact mechanism of transcriptional control remains to be determined, there are immediate and practical uses for the A_20_ SNP UTR sequence. The presented research originated from work developing new cloning/expression tools for *C. jejuni* which included using the G_20_ SNP UTR for driving constitutive transcription of specific genes (Uhlich et al., 2022). Additionally, there exists previously developed gene cloning and constitutive transcription systems (Karlyshev and Wren, 2005; Cameron and Gaynor, 2014). However, with the A_20_ SNP UTR a target gene can now be cloned downstream of the UTR and significantly different amounts of the gene transcribed depending on the growth conditions used, made possible by only a single nucleotide change in the UTR sequence. The ability to toggle gene transcription levels up and down while simultaneously observing the subsequent phenotypic and genotypic changes to the *C. jejuni* strain should constitute a useful research tool. While currently the switching between gene transcription levels is accomplished by varying growth conditions, it seem likely that there remains a more specific induction method to be discovered. This could potentially lead to a titratable induction model where multiple concentrations of an inducer can produce multiple levels of gene transcription. Further investigations of how the upstream UTR controls *cysM* transcription will increase the understanding of how *C. jejuni* can adapt to changes in its immediate environment as well as provide improvements to molecular research tools.

## Data Availability

The original contributions presented in this study are included in this article/supplementary material, further inquiries can be directed to the corresponding author.

## Footnotes

1
http://www.softberry.com/berry.phtml?topic=bprom&group=programs&subgroup=gfindb
